# Branched-Chain Amino Acids Catabolism Pathway Regulation Plays a Critical Role in the Improvement of Leukopenia Induced by Cyclophosphamide in 4T1 Tumor-Bearing Mice Treated With Lvjiaobuxue Granule

**DOI:** 10.3389/fphar.2021.657047

**Published:** 2021-10-25

**Authors:** Jun-sheng Tian, Hui-liang Zhao, Yao Gao, Qi Wang, Huan Xiang, Xiang-ping Xu, Sheng Huang, Dong-lan Yan, Xue-mei Qin

**Affiliations:** ^1^ Modern Research Center for Traditional Chinese Medicine, Shanxi University, Taiyuan, China; ^2^ Jiuzhitang Co. Ltd., Changsha, China; ^3^ School of Physical Education, Shanxi University, Taiyuan, China; ^4^ School of Life Science and Technology, Xi'an Jiaotong University, Xi'an, China

**Keywords:** lvjiaobuxue granule, leucopenia, metabolomics, biological targets network, branched-chain amino acids

## Abstract

**Background:** Cyclophosphamide is a common tumor chemotherapy drug used to treat various cancers. However, the resulting immunosuppression leads to leukopenia, which is a serious limiting factor in clinical application. Therefore, the introduction of immunomodulators as adjuvant therapy may help to reduce the hematological side effects of cyclophosphamide. Lvjiaobuxue granule has been widely used in the clinical treatment of gynecological diseases such as anemia and irregular menstruation. Recently, it has been found to increase the function of white blood cells, but its mechanism of action is still unclear. We aimed to reveal the mechanisms of Lvjiaobuxue granule against acute leukopenia by an integrated strategy combining metabolomics with network pharmacology.

**Methods:** Subcutaneously inoculated 4T1 breast cancer cells to prepare tumor-bearing mice, intraperitoneal injection of cyclophosphamide to establish a 4T1 tumor-bearing mice leukopenia animal model, using pharmacodynamic indicators, metabolomics, network pharmacology and molecular biology and other technical methods. To comprehensively and systematically elucidate the effect and mechanism of Lvjiaobuxue granule in improving cyclophosphamide-induced leukopenia in 4T1 tumor-bearing mice.

**Results:** Lvjiaobuxue granule can improve the blood routine parameters and organ index levels of the leukopenia model of 4T1 tumor-bearing mice. Metabolomics studies revealed that 15 endogenous metabolites in the spleen of mice were considered as potential biomarkers of Lvjiaobuxue granule for their protective effect. Metabonomics and network pharmacology integrated analysis indicated that Lvjiaobuxue granule exerted the leukocyte elevation activity by inhibiting the branched-chain amino acids (BCAAs) degradation pathway and increasing the levels of valine, leucine and isoleucine. The results of molecular biology also showed that Lvjiaobuxue granule can significantly regulate the key enzymes in the catabolism of BCAAs, which further illustrates the importance of BCAAs in improving leukopenia.

**Conclusion:** Lvjiaobuxue granule exerts obvious pharmacological effects on the leukopenia model of 4T1 tumor-bearing mice induced by cyclophosphamide, which could be mediated by regulating the branched-chain amino acid degradation pathway and the levels of valine, leucine and isoleucine.

## Introduction

Cyclophosphamide is the most common cancer chemotherapy agent and used in anticancer for various types of cancer, especially breast cancer (Futamura et al., 2017; Hung et al., 2017). Unfortunately, immunosuppression induced by cyclophosphamide causes the occurrence of leukopenia, which is a seriously limiting factor in a clinical application (Deng et al., 2018; Sun et al., 2018). Therefore, introducing immunomodulatory agents as supportive therapy might be useful in alleviating the hematotoxicity side effects of cyclophosphamide.

**GRAPHICAL ABSTRACT:**
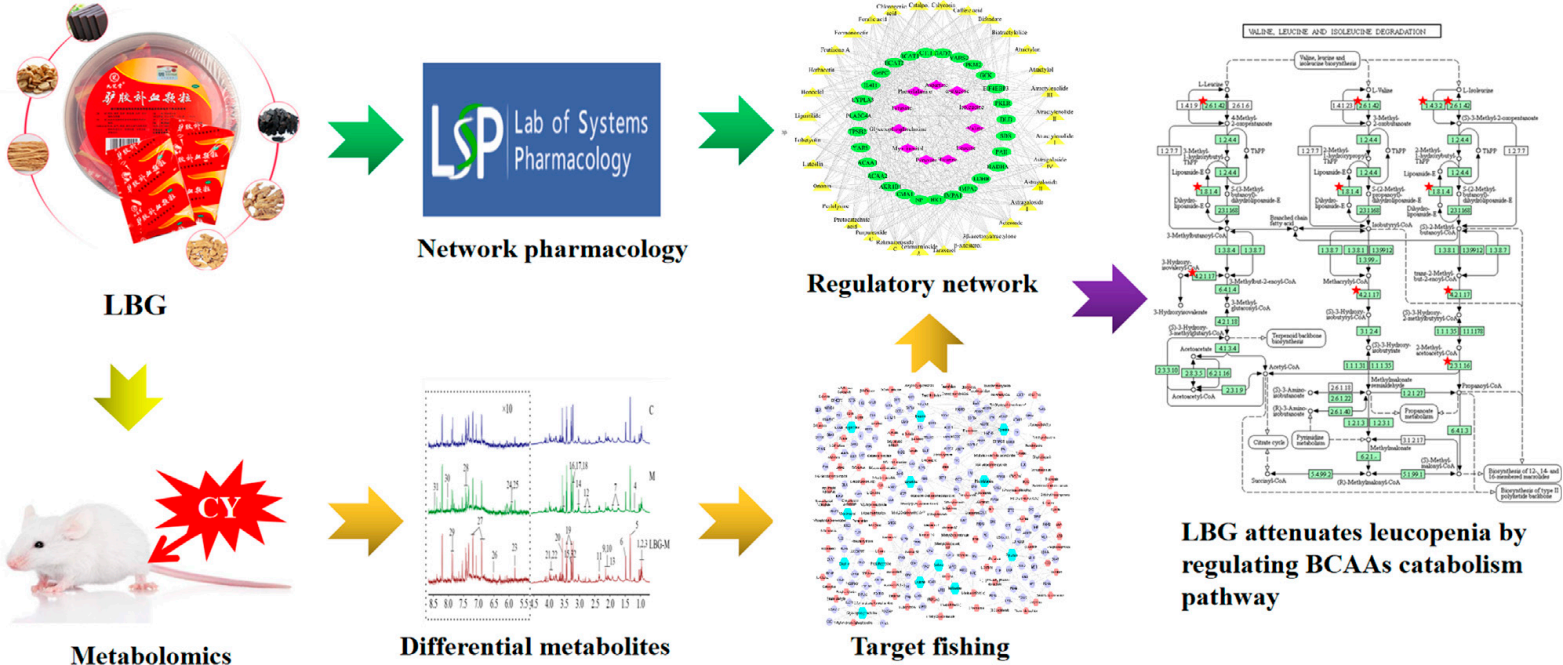


After constant efforts, the treatment against the side effects of chemotherapy still leaves much to be desired. In recent years, prescriptions are composed of a combination of multiple drugs, which are considered to be a promising treatment strategy to improve the anti-tumor effect and reduce the side effects of chemotherapy drugs (Mellor et al., 2010). Traditional Chinese Medicine (TCM) prescriptions are usually made up of different kinds of herbs, which have the advantages of low toxicity and multiple targets (Gu et al., 2014). Through overall and multi-target therapies, it has a comprehensive therapeutic effect in multi-factorial diseases.

Lvjiaobuxue granule is a new kind of good medicine for the treatment of Qi and blood deficiency and fatigue. It fills the blank of *Colla corii asini*, *Angelica sinensis* (Oliv.) Diels and other compound preparations in the treatment of deficiency of Qi and blood. It is known as the “Holy Medicine in Blood” and widely used in the clinical treatment of gynecological diseases such as anemia, irregular menstruation and so on. Besides, a large number of clinical studies have found that it has the effect of increasing white blood cells (Li and Fan, 1998; Yu, 1999; He et al., 2018). However, the research on drug pharmacological effects and mechanism of action is relatively rare. Lvjiaobuxue granule generally comprised of six herbs: *Colla corii asini* (Equidae; *Equus asinus L.* skin), *Astragalus mongholicus* Bunge (Fabaceae; *Astragalus mongholicus* radix), *Codonopsis pilosula* (Franch.) Nannf (Campanulaceae; *Codonopsis pilosula* radix), *Rehjnannia glutinosa* (Gaertn.) DC (Orobanchaceae; *Rehjnannia glutinosa* root), *Atractylodes macrocephala* Koidz (Asteraceae; *Atractylodes macrocephala* rhizoma) and *Angelica sinensis* (Oliv.) Diels (Apiaceae; *Angelica sinensis* radix) at a ratio of 36:30:30:20:15:10.

Specific immune function can be stimulated by *Colla corii asini* (Equidae; *Equus asinus L.* skin) in cyclophosphamide-induced mice (An et al., 2018). The compatibility of *Astragalus mongholicus* Bunge (Fabaceae; *Astragalus mongholicus* radix) and *Angelica sinensis* (Oliv.) Diels (Apiaceae; *Angelica sinensis* radix) [Such as Danggui-buxue Tang is the famous prescription and could increase the quantity of bone marrow mononuclear cells and peripheral blood leukocytes, enhance immunity and improve microcirculation (Zhang et al., 2019)]. Some other previous studies have also demonstrated that *Codonopsis pilosula* (Franch.) Nannf (Campanulaceae; *Codonopsis pilosula* radix), *Rehjnannia glutinosa* (Gaertn.) DC (Orobanchaceae; *Rehjnannia glutinosa* root) and *Atractylodes macrocephala* Koidz (Asteraceae; *Atractylodes macrocephala* rhizoma) exhibited effects of leukocyte elevation and immuno-enhancement (Huang et al., 2018; Tang et al., 2018). Moreover, all the above herbs in the Lvjiaobuxue granule are commonly and safely used in the formula of TCM, and rare adverse reactions were reported in clinical applications. However, the action mechanism of Lvjiaobuxue granule in the treatment of leukopenia was still poorly understood.

In recent years, metabolomics is used to clarify the scientific effects related to the effectiveness mechanism, material basis and compatibility of TCM. It will provide the technical support for the evaluation of the effectiveness of TCM, the basis of prescription substances and the understanding essence of TCM syndromes (Hu et al., 2018; Wang et al., 2018; Sun et al., 2019). However, traditional metabolomics could only reflect the terminal variation of disease and treatment (Simón-Manso et al., 2013; Singh et al., 2017). Network pharmacology as an alternative system-level method to find new drug candidates, through the entire drug-disease network, looking for multi-target drugs to reduce side effects. Network pharmacology alone can only predict the possibility of compound-target combinations and pathway analysis (Gertsch, 2011; Zhong et al., 2018). Therefore, we integrated metabolomics with network pharmacology. Metabolomics was applied to determine the influences of Lvjiaobuxue granule on leukopenia and to identify the essential metabolites. Subsequently, network pharmacology was used to analyze the proteins and reactions that regulate metabolites, as well as the targets of Lvjiaobuxue granule. In general, this strategy made up for the lack of experimental verification in network pharmacology and the lack of upstream molecular mechanisms and drug binding targets in metabolomics. This strategy will hopefully contribute to a better understanding of the therapeutic principle of natural compounds for the treatment of leukopenia.

In this study, we firstly constructed a cyclophosphamide induced leukopenia model in 4T1 tumor-bearing mice, and then used pharmacodynamic indicators, metabolomics, network pharmacology, and molecular biology techniques to comprehensively and systematically clarify Lvjiaobuxue granule improve the effect and mechanism of cyclophosphamide-induced leukopenia in 4T1 tumor-bearing mice.

## Materials and Methods

### Materials

Cyclophosphamide was obtained from Jiangsu Shengdi Pharmaceutical Co., Ltd (Batch No. 18051125, Jiangsu, China). The 0.5 ml EDTA-K_2_ anticoagulant tube and DMEM high-glucose medium and EDTA trypsin were purchased from Boshide Bioengineering Co., Ltd. Fetal bovine serum was obtained from Shanghai ShengGong Biological Engineering Co., Ltd. D_2_O was purchased from Norell (Landisville, United States). K_2_HPO_4_·3H_2_O and NaH_2_PO_4_·2H_2_O were obtained from Wuhan Yuancheng Technology Development Co., Ltd (Wuhan, China). Lvjiaobuxue granule was purchased from Jiuzhitang Co., Ltd (Batch No. 201802029, Hunan, China), which production method conforms to the standard of Chinese Pharmacopoeia (2015 edition). Diyu-shengbai tablets were obtained from Chengdu Diao Co., Ltd (Batch No. Z20026497, Chengdu, China). The enzyme-linked immunosorbent assay (ELISA) kits were obtained from MEIMIAN (Jiangsu, China).

### Animals and Cell Lines

Inbred strain female (6–8 weeks old) BALB/c mice, weighing 18–20 g, provided by Vital River Laboratory Animal Technology Co., Ltd (Beijing) (License number SCXK-2016–0006). The animals were kept at room temperature (24 ± 1)°C, humidity (60 ± 5)%, and freely eating under the natural rhythm of day and night before the experiments. The murine mammary 4T1 cancer cells were supported by Cell Bank of Shanghai, Chinese Academy of Science (CAS, Shanghai, China). All animal experiments were conducted following the NIH Guidelines for Care and Use of Laboratory Animals (U.S.A) and the Prevention of Cruelty to Animals Act (1986) of China, and all experimental procedures tried to minimize the suffering of experimental animals. The animal use protocol listed below has been reviewed and approved by the Committee of Scientific Research at Shanxi University (CSRSX), and the approved number was SXULL2018012.

### Establishment and Treatment of Leukopenia Model in 4T1 Tumor-Bearing Mice

**FIGURE 1:**
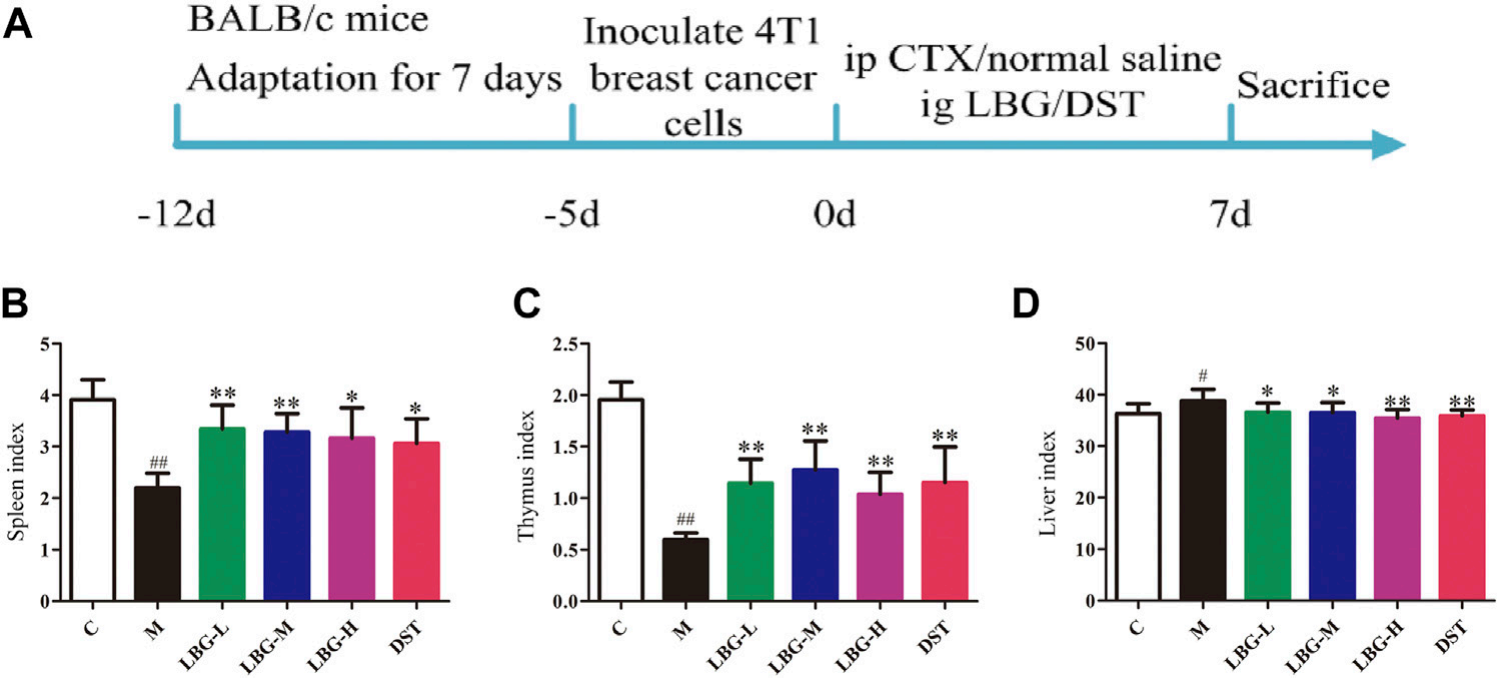
Animal modeling flow chart and the effects of LBG on organ indexes (*n* = 8). **(A)** Animal modeling flow chart. **(B)** Spleen index. **(C)** Thymus index. **(D)** Liver index. Data are expressed as mean±SD. ^#^ *p* < 0.05 *vs.* C group; ^##^ *p* < 0.01 *vs.* C group; *p < 0.05 *vs.* M group; **p < 0.01 *vs.* M group.

Animals were randomly divided into six cyclophosphamide-induced groups: control group (C), model group (M), low dose of Lvjiaobuxue granule group (LBG-L), moderate dose of Lvjiaobuxue granule group (LBG-M), high dose of Lvjiaobuxue granule group (LBG-H), and Diyu-shengbai tablets group (DST), each group was consist of eight mice. Except control group, the mice in other groups were first inoculated with breast cancer cells under the armpits to establish a tumor model, and then cyclophosphamide was given to establish a leukopenia model. Five days after mice were inoculated with 4T1 cells on the right armpit (10^7^ cells per animal), all mice developed subcutaneous tumors. On the first, third, fifth and seventh day, the model of leukopenia in 4T1 tumor bearing mice was established by intraperitoneal injection of 80 mg/kg cyclophosphamide. The mice in LBG-L, LBG-M, or LBG-H groups were administered Lvjiaobuxue granule (3 g/kg, 6 g/kg, 12 g/kg) suspension daily, and the mice in the DST group were administered Diyu-shengbai tablets (0.14 g/kg) suspension daily. Mice in the control and model groups received an equal volume of vehicle orally. During the whole experiment, the drug was administered while modeling. The treatment lasted for 7 days, and the state of the mice was observed daily (Figure 1A).

### Sample Collection and Determination

After 1 h of the last treatment on the seventh day, 0.4 ml blood samples were collected into a 1.0 ml tube with EDTA within *via* the orbital blood. Animal blood analyzer (HEMAVET950) was applied to evaluate peripheral blood routine parameters of 400 μl whole blood: white blood cell count (WBC), neutrophil count (NE), lymphocyte count (LY), monocytes count (MO), red blood cell count (RBC), hemoglobin (HGB), red blood cell volume (HCT), platelet count (PLT), mean red cell volume (MCV) and mean corpuscular hemoglobin (MCH). On the eighth day, mice were sacrificed, and spleen, thymus and liver tissues were immediately weighed and collected. The calculation formula of the organ index is as follows: organ index = organ weight (mg)/body weight (g). The spleen was quickly transferred to a refrigerator at −80°C and used for metabolomics analysis.

### Sample Preparation for NMR Measurements

After thawing the spleen tissue, took about 40 mg, cut it (on ice), added 650 μl of MeOH and H_2_O (v/v, 2:1) to a 2 ml centrifuge tube, and homogenized and extracted twice on the ice bath. The homogenate centrifuged at 4°C, 13,000 r·min^−1^ for 15 min. The supernatants were combined, transferred to a 2 ml centrifuge tube and blown with nitrogen. The dried sample was dissolved in 700 μl of phosphate buffer (pH 7.40, containing D_2_O, 0.1 mol/L, Na_2_HPO_4_/NaH_2_PO_4_, 0.01% TSP), centrifuged at 13°C, 13,000 r·min^−1^ for 20 min, 600 μl supernatant was transferred into a 5 mm NMR tube for ^1^H-NMR analysis.

### Metabolomics Analysis

The ^1^H-NMR spectral data were collected on a Bruker 600 MHz AVANCE III NMR spectrometer (Bruker, Germany). The sample was the Noesygppr 1 day sequence to suppress the water peak. The number of scans was 64 scans, and each scan required an acquisition time of 2.654 s. The specific parameters were as follows: spectral width was 12 345.7 Hz; spectrum size was 65 536 data points; pulse width (PW) was 30° (12.7 µs); fourier transform was 0.3 Hz and relaxation delay time was 1.0 s. The ^1^H-NMR spectrum of the spleen was corrected for chemical shifts using TSP (δ0.00) as the standard. The spectrum in the region of δ0.60 to 9.49 was divided into 0.01 equal widths and integrated. All resulting integration data are “mass” normalized to eliminate weight differences of spleen tissue.

### Statistical Analysis

Simca-P 14.1 (Umetrics, Sweden) was used to perform multivariate data analysis. Firstly, by principal component analysis (PCA) of the normalized data, to identify the degree of dispersion between the control group and the model group, and the outliers were eliminated. Next, partial least-squares discriminant analysis (PLS-DA) was used to distinguish the differences in metabolic profiles among the control, model and drug groups. Orthogonal-projection to latent structure-discriminant analysis (OPLS-DA) was used to find differential metabolites between the control group and the model group. Finally, GraphPad Prism 7 (GraphPad Software, Inc., La Jolla, CA, United States) and SPSS 21.0 (SPSS Software, Inc., Chicago, IL,United States) were used for statistical analysis by two-way ANOVA and Dunnett multiple comparisons. VIP>1 and *p* < 0.05 were used as differential metabolites.

The data were expressed as the mean±standard deviation (SD) of the mean from at least three independent experiments. *p* < 0.05 was considered to be statistically significant.

### Biological Targets Network Analysis

All components of Lvjiaobuxue granule were collected from the TCMSP database (https://tcmspw.com/tcmsp.php, Version 2). For all ingredients, the initial structure formats (e.g., mol2 and SDF) were transformed into a unified SDF format using the Open Babel toolkit (version 2.4.1). The ingredients with suitable OB ≥ 30%, DL ≥ 0.18 and active or higher contents in the drugs reported in the literature were chosen as candidate ingredients for further research, which were used as a selection criterion for the ingredients in the TCM. All databases and software mentioned above are public.

The PharmMapper server (http://www.lilab-ecust.cn/pharmmapper/) was used for potential target prediction analysis. Metabolite data were imported in Metascape, a plugin of Cytoscape 3.7.1 and subjected to metabolic enzyme analysis. Finally, Cytoscape 3.7.1 software was used to construct the “herb-chemical components-targets-pathway-metabolite” regulatory network of Lvjiaobuxue granule for leukopenia treatment.

### Determination of the Levels of BCKDHA and ACADS

To further verify the results of biological targets network, the content of the key rate-limiting enzymes BCKDHA (branched-chain keto acid dehydrogenase E1, alpha polypeptide) and ACADS (acyl-CoA dehydrogenase short chain) on the BCAAs degradation pathway were determined. The content of these enzymes will affect the levels and degradation rate of BCAAs. The levels of BCKDHA and ACADS in the liver tissue lysates were determined by ELISA kits according to manufacturer instructions.

## Results

### Quality Control of Lvjiaobuxue Granule

To ensure the quality control of Lvjiaobuxue granule used in this study, High Performance Liquid Chromatography (HPLC) analysis was performed. The preparation of Lvjiaobuxue granule chemical determination was referred to China Pharmacopoeia (2020 version). The HPLC system consisted of a Dual-Gradient Analytical LC System (UltiMate 3000, Dionex, United States) equipped with DGP-3600SD Pump, SRD-3600 Degasser, WPS-3000SL Autosampler, TCC-3000RS Column Compartment, DAD-3000 Diode Array Detector and Sedex-75 Evaporative Light-Scattering Detector (ELSD). Peak areas were calculated with Chromeleon 6.8 Chromatography Data System. KromasiL C18 (250 mm × 4.6 mm, 5 μm). Acetonitrile-water (40:60) was used as the mobile phase. The Carrier-gas velocity was 1.0 ml min^−1^ and the temperature of drift tube was 45°C. Taking Astragaloside IV as an example, the identified results were shown in Supplementary Figure S1.

### Effect of Lvjiaobuxue Granule on Blood Routine Parameters and Organ Indexes of 4T1 Tumor-Bearing Mice Induced by Cyclophosphamide

The biochemical indexes of the peripheral blood were observed by evaluating the blood toxicity of cyclophosphamide. The parameters of WBC, NE, LY, MO, RBC, HGB, HCT, and MCH in the model group were significantly lower than those in the control group (*p* < 0.01; Table 1), and the PLT parameters were significantly higher than those in the control group (*p* < 0.05; Table 1). The increase and decrease of these peripheral blood routine parameters (especially WBC less than 2.0*10^9^/L, NE significantly reduced) were the main diagnostic criteria of leukopenia, indicating that the model of leukopenia was successfully replicated. Compared to the model group, the expressions of WBC, NE, LY, MO, RBC, HGB, and MCH in the LBG-L, LBG-M and LBG-H were significantly increased (*p* < 0.05; Table 1). Compared to the control group, the mice in the model group had an enlargement of the liver (*p* < 0.05; Figure 1D), and reduction of the spleen (*p* < 0.01; Figure 1C) and thymus (*p* < 0.01; Figure 1C). The administration of Lvjiaobuxue granule and DST over 7 days reversed the viscera lesions of mice with hematopoietic dysfunction (*p* < 0.05; Figure 1).

**TABLE 1:** Changes in indexes of blood routine examination on leukopenia mice with LBG treatment (*n* = 8).

Groups	WBC (10^9^/L)	NE (10^9^/L)	LY (10^9^/L)	MO (10^9^/L)	RBC (10^12^/L)
C	4.63 ± 0.58	0.52 ± 0.11	3.61 ± 0.42	0.18 ± 0.04	9.99 ± 0.43
M	1.83 ± 0.25^##^	0.25 ± 0.04^##^	1.45 ± 0.21^##^	0.13 ± 0.04^#^	8.01 ± 0.23^##^
LBG-L	3.03 ± 0.44**	0.54 ± 0.12**	2.24 ± 0.35**	0.25 ± 0.04**	8.69 ± 0.18**
LBG-M	3.33 ± 0.48**	0.55 ± 0.14**	2.41 ± 0.38**	0.36 ± 0.10**	8.63 ± 0.12**
LBG-H	2.60 ± 0.20**	0.41 ± 0.16*	1.88 ± 0.12**	0.31 ± 0.09**	8.44 ± 0.18**
DST	2.79 ± 0.36**	0.36 ± 0.14*	2.08 ± 0.31**	0.30 ± 0.09**	8.00 ± 0.29

Groups	HGB (g/L)	HCT (%)	PLT (10^9^/L)	MCV (fL)	MCH (pg)
C	149.50 ± 2.78	58.69 ± 1.36	539.13 ± 54.19	58.66 ± 0.64	15.56 ± 0.37
M	124.50 ± 2.56^##^	49.50 ± 2.32^##^	615.75 ± 52.30^#^	58.89 ± 0.40	14.28 ± 0.15^##^
LBG-L	135.00 ± 5.93**	52.11 ± 2.08*	605.63 ± 40.60	59.08 ± 0.48	15.59 ± 0.24**
LBG-M	132.75 ± 1.04**	51.39 ± 1.03	602.25 ± 22.28	59.35 ± 0.61	15.69 ± 0.24**
LBG-H	130.25 ± 2.82**	49.1 ± 1.59	622.13 ± 47.92	59.68 ± 0.53	15.79 ± 0.14**
DST	128.88 ± 5.06	50.09 ± 2.25	612.38 ± 58.24	60.16 ± 0.59	15.60 ± 0.33**

Data are expressed as mean±SD. of eight mice. WBC, white blood cell count; NE, neutrophil count; LY, lymphocyte count; MO, monocytes count; RBC, red blood cell count; HGB, hemoglobin; HCT, red blood cell volume; PLT, platelet count; MCV, mean red cell volume; MCH, mean corpuscular hemoglobin. ^#^ *p* < 0.05 *vs.* C group; ^##^ *p* < 0.01 *vs.* C group; *p < 0.05 *vs.* M group; **p < 0.01 *vs.* M group.

### ^1^H-NMR Metabolomics Analysis and Multivariate Data Analysis

#### Analysis of ^1^H-NMR Spectrum of Spleen Tissue

**FIGURE 2:**
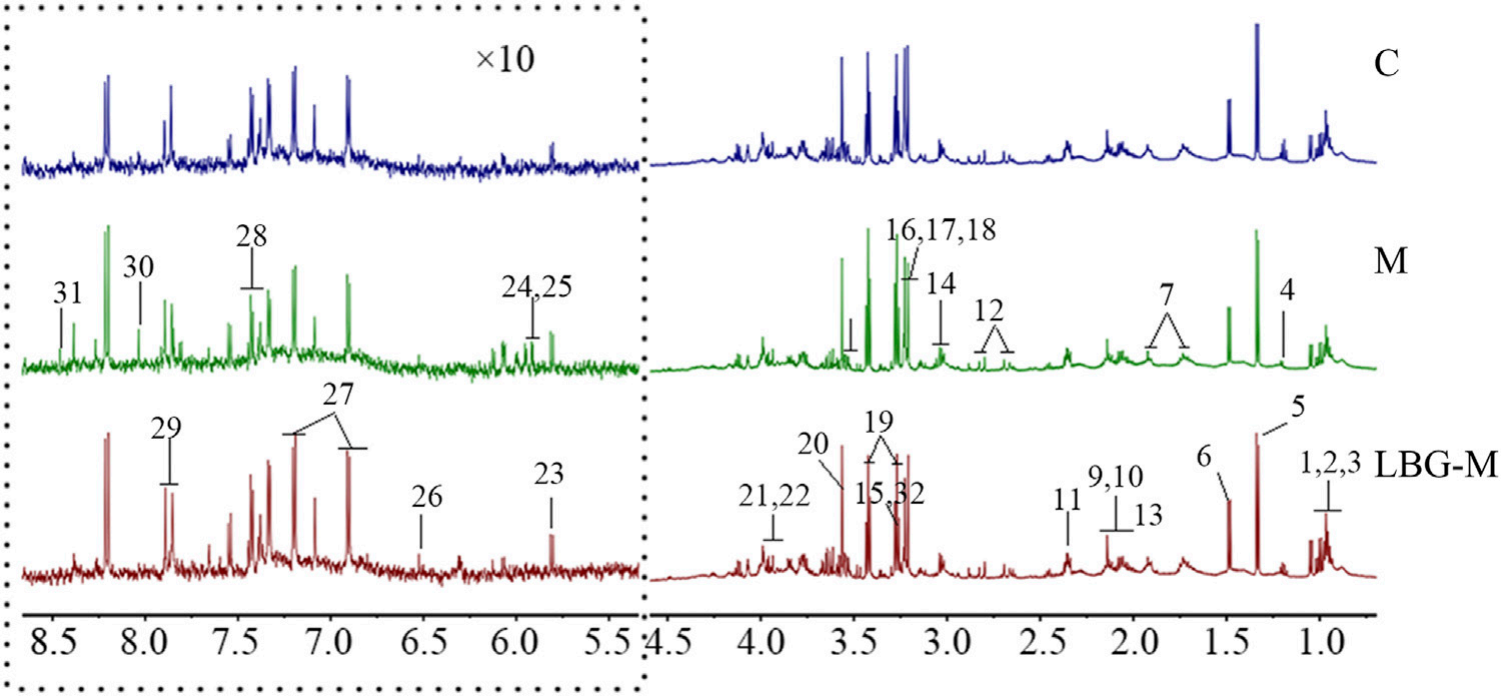
The ^1^H-NMR spectra of mice spleen tissue in C, M and LBG-M groups.

The representative ^1^H-NMR spectra of the extracts from spleen tissue of mice in control group, model group and LBG-M group were shown in Figure 2. Firstly, the ^1^H-NMR data were analyzed by chemical shift values, coupling constants and peak splitting conditions, and further identified by Human Metabolome Database [HMDB (http://www.hmdb.ca/)], Biological Magnetic Resonance Data Bank [BMRB (http://www.bmrb.wisc.edu/)] and published literature. In total, 32 endogenous metabolites were identified, belonging to amino acids, organic acids, sugars, lipids, etc. the details of the metabolites were shown in Table 2.

**TABLE 2:** ^1^H-NMR assignments of major metabolites from mice spleen tissues.

No	Metabolites	Components assignment	Chemical shift (δ ^1^H ppm)
1	Isoleucine	*δ*-CH_3_, *γ*′-CH_3_, *γ*-CH_2_, *γ*-CH_2_’, *β*-CH, *α*-CH	0.94(t), 1.01(d), 1.27(m), 1.47(m), 1.98(m), 3.68(d)
2	Leucine	*δ*-CH_3_, *δ*′-CH_3_, *β*-CH_2_, *γ*-CH, *β*-CH_2_′, *α*-CH	0.96(d), 0.97(d), 1.70(m), 1.72(m), 1.74(m), 3.74(m)
3	Valine	*γ*-CH_3_, *γ*′-CH_3_, *β*-CH, *α*-CH	0.99(d), 1.05(d), 2.28(m), 3.62(d)
4	3-hydroxybutyrate	*γ*-CH_3_, *α*-CH_2_, *α*-CH_2_′, *β*-CH	1.20(d), 2.31(dd), 2.41(dd), 4.16(m)
5	Lactate	*β*-CH_3_, *α*-CH	1.33(d), 4.11(q)
6	Alanine	*β*-CH_3_, *α*-CH	1.48(d),3.78(d)
7	Lysine	*γ*-CH_2_, *γ*-CH_2_′, *δ*-CH_2_, *β*-CH_2_, ε-CH_2_, CH	1.45(m), 1.51(m), 1.73(m), 1.91(m), 3.03(t), 3.76(t)
8	Glutamate	*β*-CH_2_, *β*-CH_2_′, *γ*-CH_2_, CH	2.07(m), 2.13(m), 2.35(m), 3.76 (dd)
9	Methionine	*δ*-CH_3_, *β*-CH_2_, *γ*-CH_2_, *α*-CH	2.13(s), 2.14(m), 2.64(t), 3.85(m)
10	Glutamine	*β*-CH_2_, *γ*-CH_2_, CH	2.14(m), 2.46(m), 3.78(t)
11	Pyruvate	CH_3_	2.37(s)
12	Aspartate	CH_2_, CH_2_′, CH	2.68(dd), 2.82(dd), 3.90(dd)
13	Acetate	CH_3_	1.93(s)
14	Creatine	CH_3_, CH_2_	3.04(s), 3.94(s)
15	Myo-inositol	2-CH, 4/6-CH, 1/3-CH, 5-CH	3.29(t), 3.54(dd), 3.63(dd), 4.07(t)
16	Choline	(CH_3_)_3_, N-CH_2_, OH-CH_2_	3.21(s), 3.52(m), 4.07(m)
17	Phosphocholine (PC)	CH_3_, N-CH_2_, O-CH_2_	3.22(s), 3.59(m), 4.18(m)
18	Glycerophosphocholine (GPC)	CH_3_, OH-CH_2_, N-CH_2_, OH-CH, NCH_2_CH_2_	3.23(s), 3.68(m), 3.68(m), 3.92(m), 4.33(m)
19	Taurine	S-CH_2_, N-CH_2_	3.28(t), 3.43(t)
20	Glycine	CH_2_	3.57(s)
21	*α*-glucose	4-CH, 2-CH, 3-CH, CH_2_, CH_2_′, 5-CH, 1-CH	3.41(m), 3.59(m), 3.73(m), 3.73(m), 3.85(m), 3.85(m), 5.26(d)
22	*β*-glucose	2-CH, 3/5-CH, CH_2_, CH_2_′, 1-CH	3.29(m), 3.52(m), 3.74(m), 3.91 (dd), 4.66 (d)
23	Uracil	5-CH, 6-CH	5.81(d), 7.55(d)
24	Uridine	ribose-2-CH, uracil-C-CH, uracil-N-CH	5.90(d), 5.92(d), 7.88(d)
25	Cytidine	ribose-2-CH, ring-5-CH, ring-6-CH	5.92(d), 6.07(d), 7.85(d)
26	Fumarate	CH	6.51(s)
27	Tyrosine	CH_2_, CH_2_′, N-CH, 3/5-CH, 2/6-CH	3.06(dd), 3.19(dd), 3.94(dd), 6.90(m), 7.20(m)
28	Phenylalanine	CH_2_, CH_2_′, N-CH, *o*-CH, *p*-CH, *m*-CH	3.13(dd), 3.28(dd), 4.00(dd), 7.33(m), 7.38(m), 7.43(m)
29	Xanthine	CH	7.90(s)
30	Hypoxanthine	2-CH, 7-CH	8.20(s), 8.22(s)
31	Formate	HCOOH	8.46(s)
32	Trimethylamine-N-oxide (TMAO)	CH_3_	3.28(s)

#### Lvjiaobuxue Granule Regulates Metabolic Disorders of Leukopenia Induced by Cyclophosphamide in 4T1 Tumor-Bearing Mice

**FIGURE 3:**
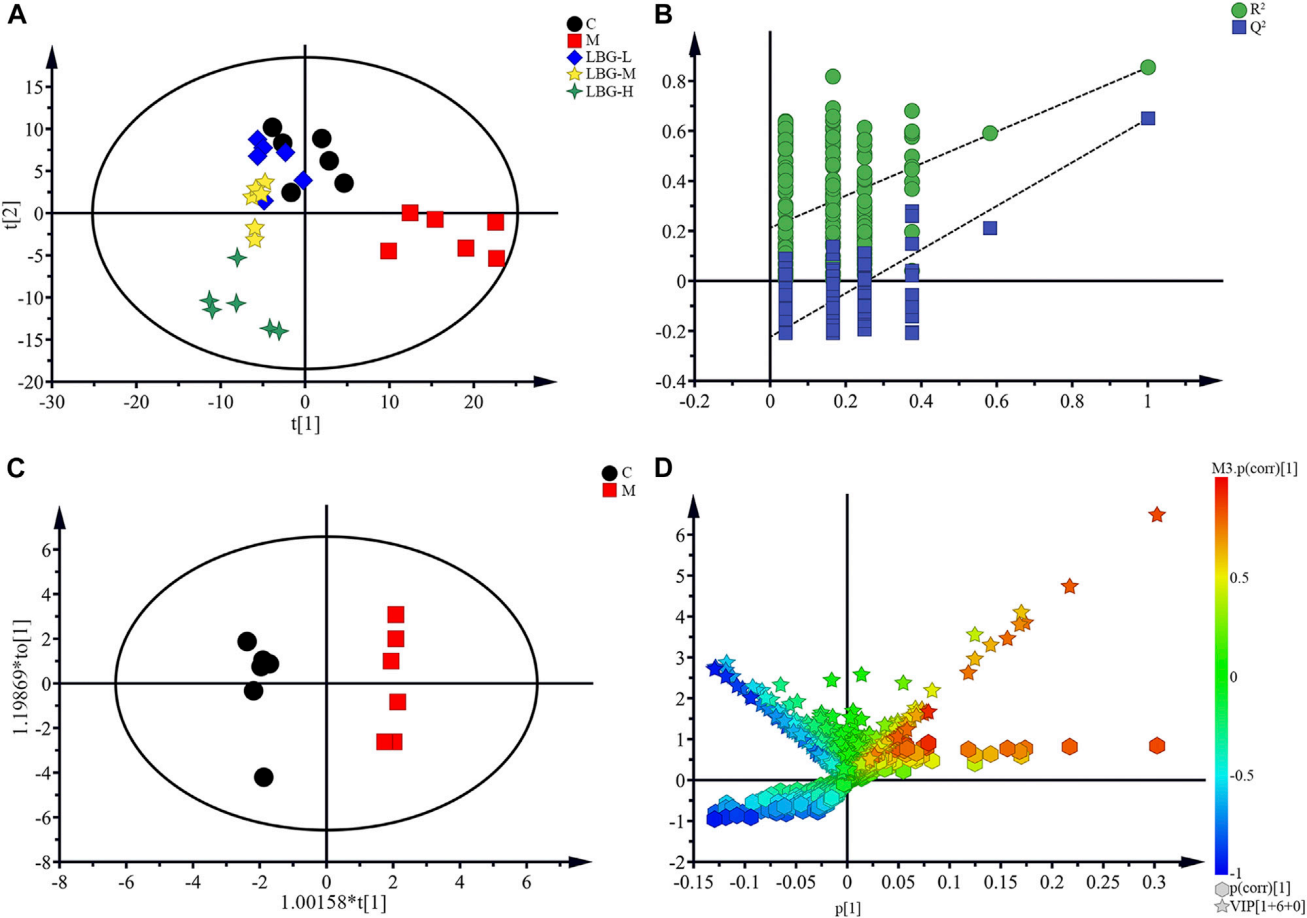
Pattern recognition of Simca-P 14.1. **(A)** Results of multiple pattern recognition of spleen metabolites in C, M, LBG groups. PLS-DA score plot (*n* = 6). **(B)** Results of permutation tests. *R* ^2^ = 0.854, Q^2^ = 0.649. **(C)** OPLS score plot (*n* = 6, R^2^Y = 0.947, R^2^X = 0.431, Q^2^ = 0.659) of C group and M group. **(D)** OPLS (S + V)-plot of C group and M group. Each point in the (S + V)-plot represents an ion. Ions far away from the origin were potential biomarkers.

To explore the metabolites levels in 4T1 tumor-bearing mice with leukopenia induced by cyclophosphamide, the supervised multivariate methods PLS-DA and OPLS-DA were used for processing (Figure 3). The PLS-DA pattern recognition analysis of all the group trends was shown in Figure 3A, in which the control group was completely separated from the model group, and the LBG-L, LBG-M, LBG-H groups were separated from the model group, with a tendency closer to the control group. It was suggested that CTX caused the disorder of mice spleen metabolites. After administration of LBG, the metabolic profile of the mice’s spleen was significantly close to that of the control group, suggesting that LBG may exert a whitening effect by adjusting the metabolites of the spleen.

The validity of the analysis was performed by using 200 permutation tests, in which all *R* ^2^ and Q^2^ values were lower than the original ones (Intercepts: *R* ^2^ = 0.854, Q^2^ = 0.649) (Figure 3B). Furthermore, differential metabolites between the control and the model groups were discovered by OPLS-DA, which was a supervised pattern recognition method that could improve the discovery effect of differential metabolites (Figure 3C). The corresponding loading (S + V)-plot with color-coded was illustrated in Figure 3D, and the metabolites contributed to the leukocyte elevation effect were identified by corresponding (S + V)-plots and statistical analysis.

**FIGURE 4:**
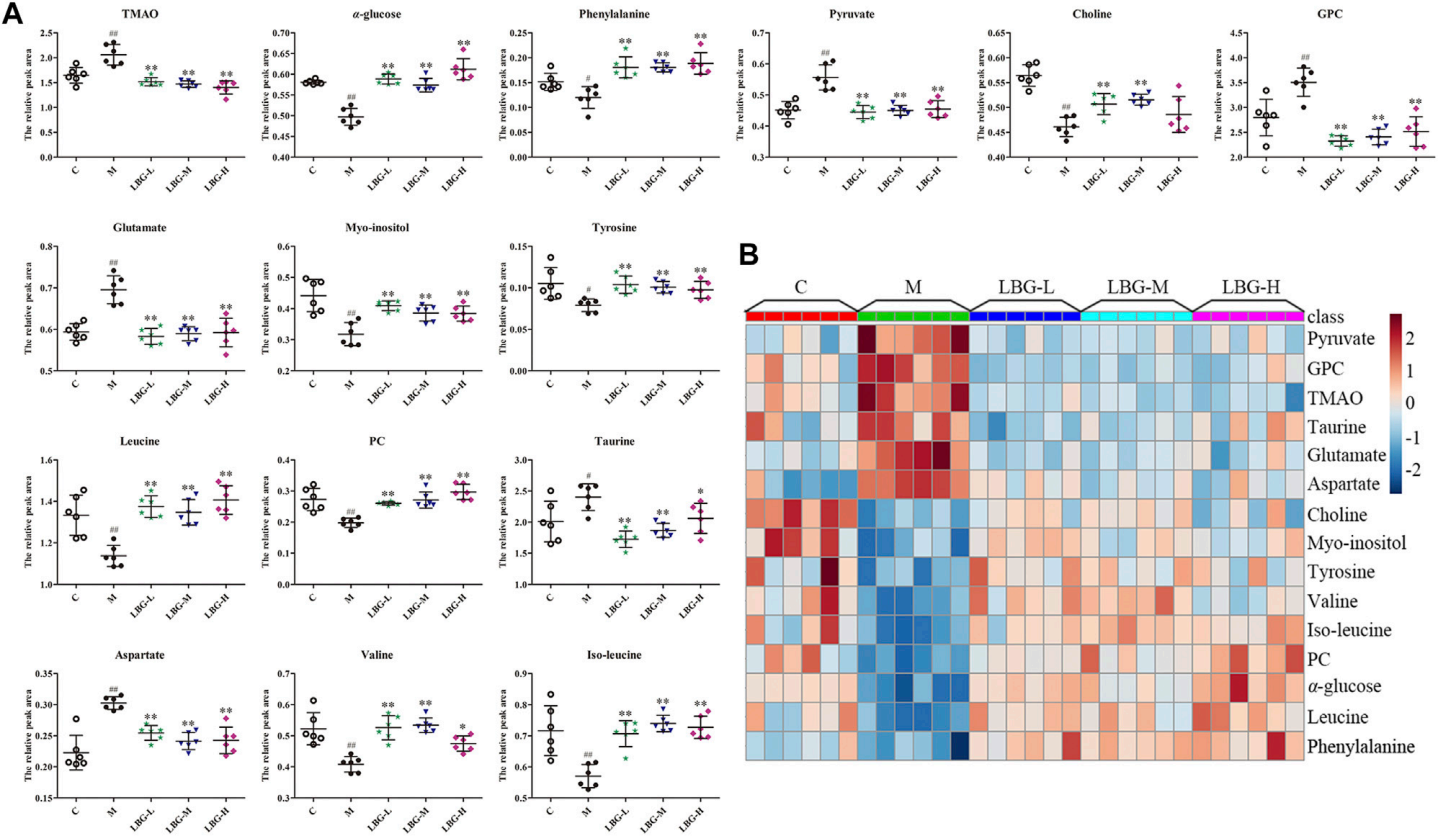
**(A)** Box-plots of the changes of differential metabolites of mice spleens in C, M and LBG groups. Data are expressed as mean±SD. ^#^ *p* < 0.05 *vs.* C group; ^##^ *p* < 0.01 *vs.* C group; *p < 0.05 *vs.* M group; **p < 0.01 *vs.* M group. **(B)** The relative content of the heatmap of differential metabolites in mice spleens. The ribbon −3∼3: represents the relative content of the differential metabolites from low to high.

The disturbed metabolite variances of the different groups could be related to the decrease and increase of leukocytes. The changes of the differential metabolites between the control and the model groups in mice spleen were shown in Figure 4. Compared with the control group, the elevated levels of TMAO, pyruvate, GPC, taurine, aspartate and glutamate in the model group were evident in the spleen samples. Additionally, lower levels of choline, myo-inositol, tyrosine, valine, iso-leucine, PC, *α*-glucose, leucine, and phenylalanine in the spleen of the model group were observed. The changes of these endogenous metabolites were considered to be a direct result of the leukopenia. Meaningfully, the levels of metabolites were regulated by LBG treatment (Figure 4), suggesting that LBG may play a role in leukocyte by adjusting the level of metabolites.

#### Correlation Analysis of Differential Metabolites, Blood Routine Parameters and Organ Indexes

**FIGURE 5:**
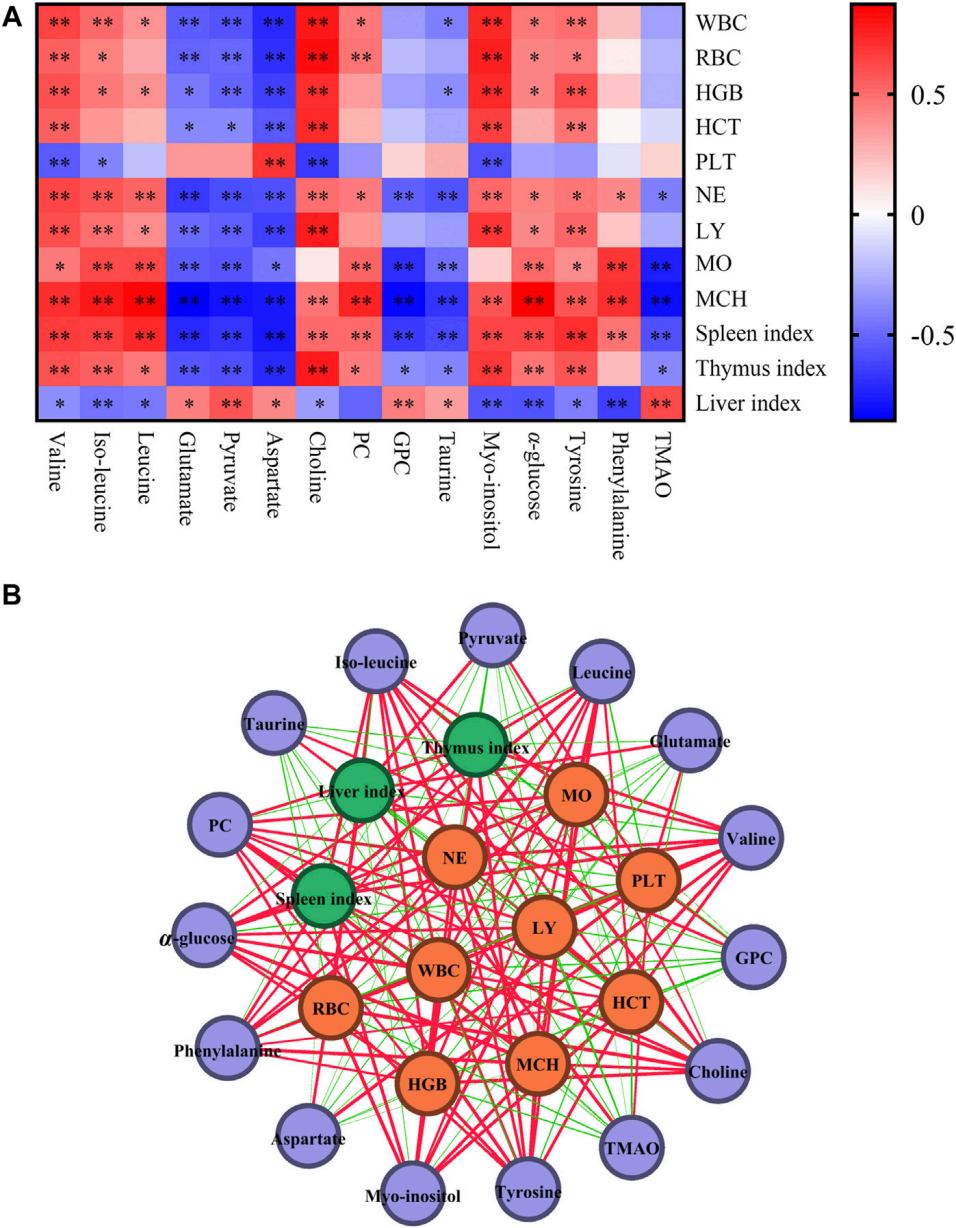
Correlation analysis of differential metabolites, blood routine parameters and organ indexes. **(A)** The correlation map of differential metabolites, blood routine parameters and organ indexes. Red and blue represent positive and negative correlations respectively (*p < 0.05, **p < 0.01). **(B)** Correlation network of differential metabolites, blood routine parameters and organ indexes based on Pearson’s correlation coefficients. Purple, yellow and green nodes represent differential metabolites, blood routine parameters and organ indexes. Red and green lines represent positive and negative correlations. Line thickness reflects the magnitude of the correlation coefficients.

Blood routine parameters and organ index were used to evaluate important indicators of leukopenia. Through the correlation analysis with different metabolites, it was helpful to screen the key differential metabolites and clarify the regulatory role of different metabolites on the body. Pearson’s correlation analysis was used to visualize the different metabolites, blood routine parameters and organ index. The correlation map was shown in Figure 5A, where the color reflects the correlation strength and sign, WBC, RBC, HGB, HCT, NE, LY, MO, and MCH were negatively correlated with glutamate, pyruvate, aspartate, GPC, taurine and positive correlations with valine, iso-leucine, leucine, choline, PC, myo-inositol, *α*-glucose, tyrosine, and phenylalanine. In addition, the fluctuation of spleen index was correlated with different metabolites (*p* < 0.01) (Figure 5A). The correlation network of blood routine parameters, organ index and differential metabolites based on Pearson’s was shown in Figure 5B, which could be served as differential metabolites for assessing the leukopenia and the effect of Lvjiaobuxue granule.

#### Establishment of “Spleen Differential Metabolite-Target/Metabolic Enzyme-Related Metabolite” Network

**FIGURE 6:**
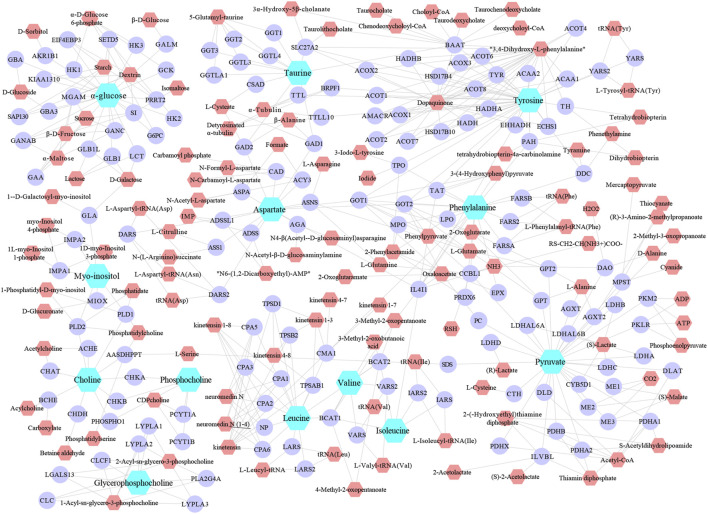
The metabolic networks involved in enzymes, genes and related-compound were established based on the differential metabolites. Light-blue, blue and pink nodes represent differential metabolites, enzymes/genes and related-compound.

The metabolic networks involved in a series of enzymes and genes were constructed by using the Metscape plug-in running on Cytoscape 3.7.1, that the internal correlation of the differential metabolites based on enzyme or gene levels could be better understood (Figure 6). As a result, 154 candidate genes or enzymes related to differential metabolites were tentatively found out, and they will be served as targets for subsequent biological targets network analysis for the construction of targets-metabolites interactions network (Figure 6). Finally, to find the metabolic pathway of identified differential metabolites from leukopenia-associated researches, the enrichment analysis was performed and metabolic pathways were obtained for further investigation.

### Network Pharmacology and Metabolomics Integration Analysis

#### Screening Active Compounds of Lvjiaobuxue Granule

In the current study, two ADME-related parameters, including OB and DL, were employed to screen for active ingredients in Lvjiaobuxue granule. After ADME screening, some ingredients that did not meet the screening criteria were selected because of their high content and high biological activity. First of all, a gross of 87, 134, 76, 55, and 125 candidate ingredients were obtained from *Astragalus mongholicus* Bunge (Fabaceae; *Astragalus mongholicuss* radix), *Codonopsis pilosula* (Franch.) Nannf (Campanulaceae; *Codonopsis pilosula* radix), *Rehjnannia glutinosa* (Gaertn.) DC (Orobanchaceae; *Rehjnannia glutinosa* root), *Atractylodes macrocephala* Koidz (Asteraceae; *Atractylodes macrocephala* rhizoma) and *Angelica sinensis* (Oliv.) Diels (Apiaceae; *Angelica sinensis* radix) respectively. The ingredients were retrieved from these ingredients *via* the ADME parameters and literature confirmation. Consequently, a total of 32 active ingredients of Lvjiaobuxue granule were filtered out for further analysis (Supplementary Table S1).

#### Construction of “Chemical Components-Targets-Differential Metabolites” Regulatory Network and Correlative Pathways

**FIGURE 7:**
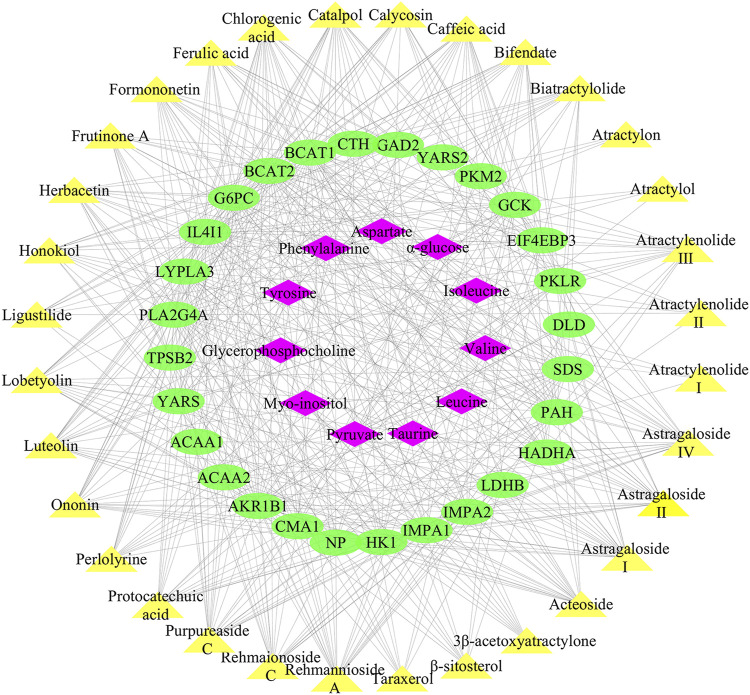
“Active ingredients-targets-differential metabolites” regulatory network of LBG. Yellow nodes represent the active ingredients, green nodes represent the targets, and pink nodes represent differential metabolites.

490 targets of the 32 active ingredients in Lvjiaobuxue granule were predicted using the PharmMapper server, and 28 of them were closely related to differential metabolites of leukopenia with a total frequency of 496 (Supplementary Table S2). Cytoscape software was used to establish the “chemical components-targets-differential metabolites” regulatory network of Lvjiaobuxue granule, which was an indication that the correlations of 32 active ingredients, 28 metabolite-associated target proteins, and 11 differential metabolites were presented in Figure 7. Analysis of the “active ingredients-targets” correlation revealed that multiple compounds could act on the same target, and multiple targets could be affected by the same compound. For instance, the L-amino-acid oxidase could be the target of ferulic acid, caffeic acid, biatractylolide simultaneously, while the compound of catalpol could act on Aldo-keto reductase family one member B1, Eukaryotic translation initiation factor 4E-binding protein 3, and Glucose-6-phosphatase at the same time (Figure 7).

To assess the molecular mechanisms of Lvjiaobuxue granule effects on leukopenia, the Cytoscape with its plugin ClueGO was utilized for KEGG pathway analysis, followed by the analysis of target-related pathway. A total of nine significant pathways were predicted (*p* < 0.05), among which the valine, leucine and isoleucine degradation were the most closely related (Table 3). According to the results of biological targets network and metabolomics, the mechanism of increasing leukocyte activity of Lvjiaobuxue granule was preliminarily predicted as follows: Lvjiaobuxue granule can inhibit the valine, leucine and isoleucine degradation and add the levels of valine, leucine and isoleucine (Figure 4A).

**TABLE 3:** The results of KEGG correlative pathways of LBG major active ingredient related targets were analyzed by Cytoscape 3.7.1 software.

Rank	KEGG term	*p*-value	Associated genes found
1	Valine, leucine and isoleucine degradation	2.76E-10	ACAA1, ACAA2, BCAT1, BCAT2, DLD, HADHA, IL4I1
2	Glycolysis/Gluconeogenesis	3.46E-9	DLD, G6PC, GCK, HK1, LDHB, PKLR, PKM
3	Cysteine and methionine metabolism	1.06E-8	BCAT1, BCAT2, CTH, IL4I1, LDHB, SDS
4	Galactose metabolism	4.41E-6	AKR1B1, G6PC, GCK, HK1
5	Pyruvate metabolism	1.13E-5	DLD, LDHB, PKLR, PKM
6	Propanoate metabolism	2.10E-4	DLD, HADHA, LDHB
7	Starch and sucrose metabolism	2.99E-4	G6PC, GCK, HK1
8	Glycine, serine and threonine metabolism	4.10E-4	CTH, DLD, SDS
9	Fatty acid degradation	5.44E-4	ACAA1, ACAA2, HADHA

#### Molecular Docking Verification

KEGG pathway analysis revealed that the decomposition pathway of valine, leucine and isoleucine was the most important way for the Lvjiaobuxue granule to improve cyclophosphamide-induced leukopenia in 4T1 tumor-bearing mice (Table 3), and then constructed the chemical component-target-differential metabolite regulatory network. Supplementary Figure S2 showed 35 compounds, seven metabolite-related target proteins, and three correlation of different metabolites. Furthermore, the SystemsDock (http://systemsdock.unit.oist.jp/iddp/home/index) method was used to evaluate the binding potential between selected valine, leucine and isoleucine degradation pathway targets and the chemical components with top 7° (Degree ≥ 5) (Supplementary Figure S2). The docking score of SystemsDock can directly indicate the protein-ligand binding potential. The 3D structures and PDB ID of the above seven selected targets were gathered from the PDB database (https://www.rcsb.org/) (Supplementary Table S3). The results showed that six targets (ACAA2, BCAT1, BCAT2, DLD, HADHA, and IL4I1) had 3D structures, while the 3D structure of ACAA1 did not exist. As shown in Supplementary Figure S3, the docking scores of 95 pairs of target-compound combinations were mostly greater than native ligand, which showed that they possessed great binding activity.

### Experimental Validation of BCAAs Catabolism Pathway by ELISA

**FIGURE 8:**
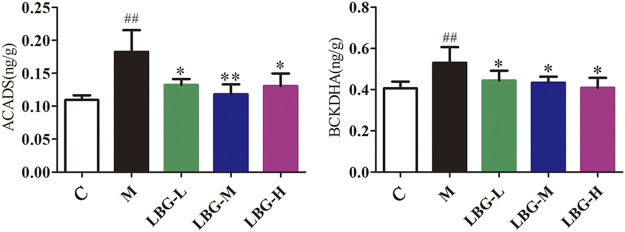
Expression of ACADS and BCKDHA enzyme levels in mice liver tissue using ELISA analysis. Data are expressed as mean±SD. ^##^ *p* < 0.01 *vs.* C group; *p < 0.05 *vs.* M group; **p < 0.01 *vs.* M group.

To further confirm the results of the biological targets network, we verified the BCAAs catabolism pathway. Two key and irreversible reactions of BCAAs catabolism require the participation of branched-chain keto acid dehydrogenase (BCKDH) complex and acyl-CoA dehydrogenase (ACAD). The levels of BCKDHA and ACADS were significantly increased (*p* < 0.01) in the model group (Figure 8). After oral administration of Lvjiaobuxue granule, the levels of BCKDHA and ACADS were significantly reduced (*p* < 0.05 or *p* < 0.01). The BCKDHA and ACADS enzyme levels in the model group were significantly increased in the catabolism pathway of BCAAs. Abnormal expression of BCAAs degrading enzymes cause BCAAs to overly decomposed in the model group. Lvjiaobuxue granule can reverse the levels of two enzymes, thereby improving the abnormal metabolism of BCAAs. In the leukopenia model group, the abnormal expression of BCAAs degrading enzyme lead to the excessive decomposition of BCAAs.

## Discussion

In this study, we observed the pharmacological effects profiles of low, moderate and high dose Lvjiaobuxue granule on the increase of leukocytes in 4T1 tumor-bearing mice model of leukopenia induced by cyclophosphamide for the first time. The major new findings were that Lvjiaobuxue granule could improve cyclophosphamide-induced leukopenia in 4T1 tumor-bearing mice, accelerate the recovery of spleen, thymus and liver indices, accelerate the recovery of WBC, NE, LY, Mo, and RBC contents, and the recovery of spleen metabolite levels in the mice spleen caused by leukopenia. Thus, the *in vivo* treatment with Lvjiaobuxue granule accelerated recovery of leukopenia after chemotherapy in mice. In this study, an integrated approach of metabolomics and network pharmacology was performed to explore the biological mechanisms of Lvjiaobuxue granule in the treatment of cyclophosphamide induced leukopenia in 4T1 tumor-bearing mice. According to the results of network pharmacology and metabolomics, that regulation on the BCAAs catabolism pathway may play a key role in cyclophosphamide induced leukopenia in 4T1 tumor-bearing mice after the treatment of a typical TCM of Lvjiaobuxue granule.

### Construction of Leukopenia Model in 4T1 Tumor-Bearing Mice and Dosage Screening of Lvjiaobuxue Granule

Cyclophosphamide, a cell cycle non-specific anti-tumor drug, is one of the most commonly used drugs for establishing an animal model of leukopenia (Huyan et al., 2011; Qu et al., 2016). The cyclophosphamide can easily block the division and proliferation of normal hematopoietic cells, lead to impaired bone marrow regeneration or damage to the hematopoietic system, decrease the number of bone marrow nucleated cells, and decrease hematopoietic reconstitution activity. Among them, leukopenia is the most common result (Fan et al., 2017; Luo et al., 2017). By combing the references, the cyclophosphamide-induced leukopenia model has been relatively mature, but the mouse species, dosage and duration are different, and it is mostly used to stimulate bone marrow suppression and immunosuppression caused by chemotherapy (Huang et al., 2017; Khan, 2017; Zhang et al., 2017; Sun et al., 2018). However, in clinical practice, the cyclophosphamide is used for chemotherapy in cancer patients, while animal models are often used in normal mice, which is different from clinical practice. Therefore, in this study, cyclophosphamide was used for the first time to induce a 4T1 tumor-bearing mice leukopenia model to simulate clinical and pathophysiology.

The research previously conducted a dose screening on the cyclophosphamide-induced leukopenia model, and found that the effect of Lvjiaobuxue granule in increasing leukocytes was not dose-dependent, and the dose of 24 g/kg Lvjiaobuxue granule caused a sharp increase in white blood cells. Exceeding the normal level, affecting the normal level of the body. Among them, 6 g/kg has the most significant effect on white blood cell improvement. Therefore, during the experiment, we chose 3 g/kg, 6 g/kg, 12 g/kg three doses (He, 2018). DST is a pure Chinese medicine preparation composed of *Sanguisorba officinalis* Linn (Rosaceae; *Sanguisorba officinalis* radix) single medicine, which can stimulate bone marrow hematopoiesis, promote the proliferation and differentiation of hematopoietic stem progenitor cells, increase the number of blood cells produced, and effectively improve the white blood cell level of leukopenia. Radiotherapy and chemotherapy can cause bone marrow suppression in cancer patients, and their hematopoietic microenvironment will also be destroyed. In addition, DST can prevent the DNA damage of bone marrow hematopoietic cells caused by radiotherapy and chemotherapy, and have the effect of protecting the hematopoietic microenvironment (Jia et al., 2012; Zhu et al., 2020). It has a good clinical effect in the treatment of leukopenia without adverse side effects.

### Blood Routine Parameters

Hematopoietic dysfunction including leukopenia, hematopoietic suppression and immunosuppression were observed in patients receiving cyclophosphamide with malignant tumors. The occurrence of leukopenia may be related to hematopoietic stem cell dysfunction, apoptosis of bone marrow cells and imbalance of hematopoietic regulatory factors (Cook and Nutini, 1971; Christen et al., 2017). Thus rebuilding hematopoietic function is the primary problem of adjuvant chemotherapy. In this study, significant decreases in WBC, NE, LY, MO, RBC, and HGB were observed through the impact of cyclophosphamide, and these altered blood routine parameters were regulated by Lvjiaobuxue granule treatment. The cyclophosphamide can degrade to amido nitrogen mustard, acrolein and so on, which bind to the DNA of rapidly growing cells and produce strong toxicity in the cells, while Lvjiaobuxue granule may play a role in leukocyte elevation through the reduction of DNA damage and improvement of bone marrow hematopoietic function (Stroda et al., 2017). In addition, the results of this study identified 15 differential metabolites in nuclear magnetic metabolomics, and the correlation analysis showed that the decreased content of differential metabolites was positively correlated with blood routine index levels, indicating that blood routine test results were correlated with metabolomics. The results were consistent, which verified the reliability of metabolomics results to a certain extent.

### Organ Indexes

As the most important immune organs in mammals, spleen and thymus are the places where immune cells grow and proliferate (Zhang et al., 2012). The developmental status of the spleen and thymus directly affects immune function and disease resistance (Duan et al., 2017; Sasaguri et al., 2017; Mahaki et al., 2019). The results of this experiment showed that the spleen and thymus indexes of the high, moderate and low dose Lvjiaobuxue granule groups were significantly higher than those of the model group. It was suggested that Lvjiaobuxue granule could resist the toxic effects of cyclophosphamide on the development of spleen and thymus. Cyclophosphamide is a commonly used chemotherapeutic drug in clinical practice. It kills tumor cells but also destroys normal cells. It is hydrolyzed by excessive phosphatases or phosphatases in the liver or tumors. Therefore, the liver is the main metabolic site of cyclophosphamide. (Lin et al., 2017; Yang et al., 2018). Moreover, the liver is the main drug metabolism organ in mammals. Metabolized cyclophosphamide causes damage to mitochondria and damage to cellular respiration, which affects the onset of lipid peroxidation and an increase in reactive oxygen species (Stankiewicz and Skrzydlewska, 2003; Olayinka et al., 2015). In this study, significant increases in the liver index were observed through the impact of cyclophosphamide, and these altered were regulated by Lvjiaobuxue granule’ treatment. Hence, Lvjiaobuxue granule may play an important role in improving liver index by reducing lipid peroxidation and reactive oxygen species production.

### Integration of Metabolomics and Network Pharmacology

Metabolomics are limited to a listing of potential metabolites and related pathways without further exploration of their direct relationships. Network pharmacology can further validate the therapeutic regulation of metabolic networks and facilitate the identification of key targets and biomarkers. The organic combination of network pharmacology and metabolomics effectively explains how Lvjiaobuxue granule can regulate metabolite levels by intervening in metabolic enzymes in the body, thereby improving the mechanism of leukopenia. KEGG analysis results shown that Lvjiaobuxue granule may improve leukopenia in mice by regulating the catabolic pathways of valine, leucine and isoleucine.

### BCAAs Catabolism

**FIGURE 9:**
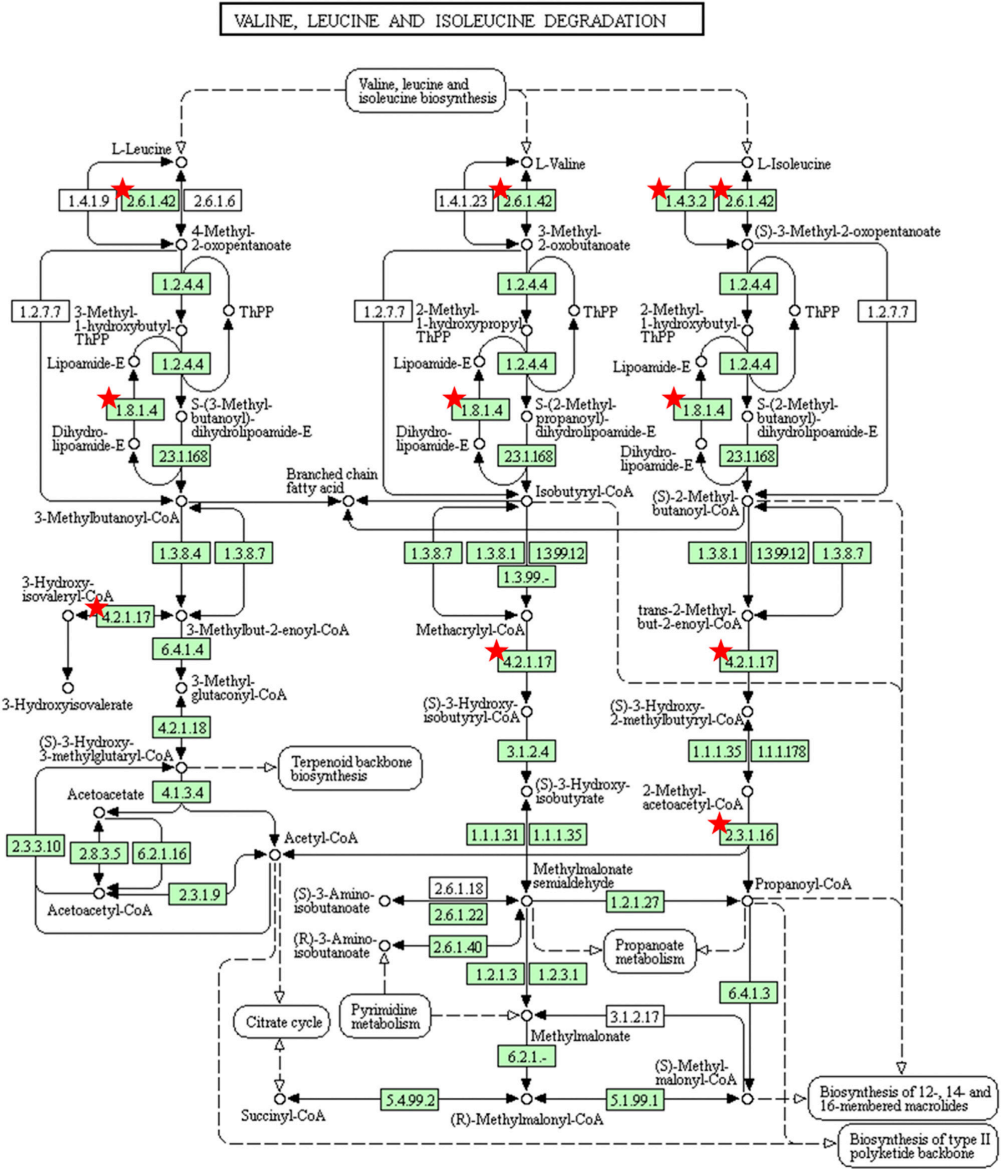
Pathway of branched-chain amino acids catabolism found by KEGG Pathway maps. The organism-specific pathways are colored green, where coloring indicates that map objects exist and are linked to corresponding entries. The red stars mark potential targets of LBG in pathways. It is indicated that Lvjiaobuxue granule attenuates cyclophosphamide-induced leukopenia by regulating branched-chain amino acids catabolism pathway.

The BCAAs are essential amino acids for human body, which cannot be synthesized *in vivo* and can only be obtained from food. BCAAs catabolism has been focused on a great deal of diseases, especially liver cirrhosis, renal failure, sepsis and cancer (Figure 9) (Holeček, 2018). Numerous studies have shown that BCAAs have a positive effect on regulating body weight, muscle protein synthesis, insulin secretion, the aging process, and prolonging the healthy period (Ikeda et al., 2018; Xu et al., 2018; Yamada et al., 2018). BCAAs have been shown to an important role in regulating body metabolism and maintaining energy balance by directly affecting body tissues, such as white adipose tissue, liver tissue and muscle tissue (Lashinger et al., 2011). BCAAs can be used as energy substrates in catabolic states, which can be directly oxidized or converted to glycogen isolipoproteins in the muscle. In contrast, BCAAs stimulate protein synthesis and cell growth in anabolic conditions (De Palo et al., 2001).

In the current study, we used 19 potential active ingredients of Lvjiaobuxue granule as a probe to conduct molecular docking with potential targets of BCAAs catabolism, and the molecular docking showed that they possessed great binding activity. A study revealed that astragaloside Ⅳ, formononetin, calycosin and ferulic acid showed promoting hematopoiesis through regulating cyclin-related proteins, promoting cell cycle transformation, and promoting HSC proliferation (Liu et al., 2015). Another study showed that caffeic acid can down-regulated the expression of TLR-2 and HLA-DR, and inhibited the production of cytokines, then exerted an immunomodulatory action on human monocytes (Búfalo and Sforcin, 2015). Studies of receiver biases suggested that the chemical components of the Lvjiaobuxue granule have a better activity of immunity regulation and hematopoiesis. The results of molecular docking showed that there were complex interactions between them, which showed the characteristics of multi-components and multi-targets. In this study, the levels of valine, leucine and isoleucine decreased significantly in the cyclophosphamide-induced mice, which was a suggestion that the leukopenia was associated with the branched-chain amino acids catabolism. Moreover, the branched-chain amino acids were positively correlated with WBC, NE, LY, and MO in the leukopenia mice, which were an indication that they played key roles in the progression of leukopenia. Compared with cyclophosphamide-induced mice, the levels of valine, leucine, and isoleucine can be improved significantly in the Lvjiaobuxue granule groups, which was a suggestion that the Lvjiaobuxue granule could play a key role in the leukocyte elevation effect by inhibiting the BCAAs catabolism. In this study, two key enzymes, the BCKDHA and ACADS enzyme levels in leukopenia, significantly increased in the BCAAs catabolism pathway. Abnormal expression of BCAAs degrading enzymes causes BCAAs overly decomposed in leukopenia model mice. Lvjiaobuxue granule can reverse the levels of two enzymes, thereby improving the abnormal metabolism of BCAAs.

Although this study had systematically explained the mechanism of Lvjiaobuxue granule, there are still the following aspects worthy of further study:

1). At the metabolite level, this study only used 1H-NMR metabolomics technology to reveal the changes in the level of branched-chain amino acids, and only detected the upstream metabolites leucine, isoleucine and valine in the catabolism of BCAAs. Unable to detect its downstream substances. 2). At the genetic level, although this study uses the method of network prediction to find out the genes that affect the changes of metabolites and the genes regulated by Lvjiaobuxue granule, it is still necessary to further determine the accuracy of the prediction results. 3). At the level of enzymes and proteins, this study only used ELISA to determine the content of the key metabolic enzymes ACADS and BCKDHA in the BCAAs catabolic pathway, but it is not known whether the enzyme activity changes when the BCAAs catabolic pathway changes. 4). At the pathway level, this study conducted a systematic study on the regulation of BCAAs from the metabolite to the molecular level. However, both KEGG pathway analysis and metabolomics results show that Lvjiaobuxue granule have effects on other pathways [amino acid metabolism (glutamine, phenylalanine), energy metabolism and choline metabolism, etc.] and metabolites (choline, tyrosine, phenylalanine, etc.) also have a certain effect. In view of the overall regulatory effect of TCM, it is not comprehensive to study only the decomposition pathway of BCAAs.

## Conclusion

In summary, we first established a model of leukopenia after chemotherapy, which can be used to study the effect of drugs on leukopenia. Determine the method and dosage of cyclophosphamide. This model can reflect the pathophysiological nature of clinical patients. Based on metabolomics and network pharmacology, a new comprehensive strategy was developed to construct an “active ingredients-targets-differential metabolites” network, which involved 32 active ingredients, 28 potential targets, and 11 differential metabolites. KEGG results showed that Lvjiaobuxue granule can improve leukopenia in mice by regulating the decomposition pathway of valine, leucine and isoleucine (BCAAs). The molecular docking results showed that the active ingredients of Lvjiaobuxue granule and the six targets on the decomposition pathway of BCAAs had a high binding force, indicating the reliability of the biological target network analysis results. Molecular biology verification results showed that Lvjiaobuxue granule can significantly reduce the key metabolic enzymes ACADS and BCKDHA in the catabolism of BCAAs, thereby playing a role in the treatment of leukopenia. This research provided data and theoretical support for in-depth study of its mechanism, and lay a foundation for clinical application.

## Data Availability

The raw data supporting the conclusions of this article will be made available by the authors, without undue reservation, to any qualified researcher.

## Abbreviations

BCAAs, Branched-chain amino acids; LBG, Lvjiaobuxue granule; TCM, Traditional Chinese medicine; DST, Diyu-shengbai tablets, WBC: white blood cell count; NE, neutrophil count; LY, lymphocyte count; MO, monocytes count; RBC, red blood cell count; HGB, hemoglobin; HCT, red blood cell volume; PLT, platelet count; MCV, mean red cell volume; MCH, mean corpuscular hemoglobin; TSP, 2,2,3,3-trimethyl siliconalkyl propionate; PW, pulse width; PCA, principal component analysis; PLS-DA, partial least-squares discriminant analysis; OPLS-DA, Orthogonal-projection to latent structure-discriminant analysis; BCKDHA, branched-chain keto acid dehydrogenase E1, alpha polypeptide; ACADS, Acyl-CoA Dehydrogenase Short Chain; TMAO, Trimethylamine-N-oxide; PC, Phosphocholine; GPC, Glycerophosphocholine.
