# Supplementary material for: Branched-Chain Amino Acids Catabolism Pathway Regulation Plays a Critical Role in the Improvement of Leukopenia Induced by Cyclophosphamide in 4T1 Tumor-Bearing Mice Treated With Lvjiaobuxue Granule
Source: Front Pharmacol. 2021 Oct 25;12:657047. doi: 10.3389/fphar.2021.657047 (PMC8573099; doi:10.3389/fphar.2021.657047)
Supplement: Supplementary file 1 [file DataSheet1.pdf]

Supplementary materials

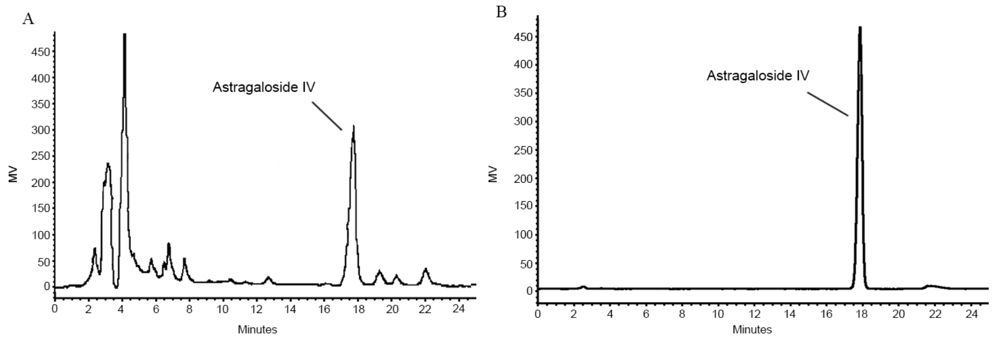

Supplementary Figure 1. Chromatograms of Lvjiaobuxue granule (LBG) (A) and Astragaloside IV (B).

Supplementary Table 1. Active ingredients and ADME parameters of formula LBG.

No.	Name	OB (%)	DL	Herb
1	Formononetin	69.67	0.21	*Astragalus membranaceus* Bunge
2	Calycosin	47.75	0.24	*Astragalus membranaceus* Bunge
3	Caffeic acid	54.97	0.05	*Astragalus membranaceus* Bunge
4	Astragaloside I	46.79	0.11	*Astragalus membranaceus* Bunge
5	Astragaloside II	46.06	0.14	*Astragalus membranaceus* Bunge
6	Bifendate	31.10	0.67	*Astragalus membranaceus* Bunge
7	Astragaloside IV	22.50	0.15	*Astragalus membranaceus* Bunge
8	Ononin	11.52	0.78	*Astragalus membranaceus* Bunge
9	Acteoside	2.94	0.62	*Astragalus membranaceus* Bunge,<br>*Rehjnannia glutinosa* (Gaertn.) DC.
10	Perlolyrine	65.95	0.27	*Codonopsis pilosula* (Franch.) Nannf.
11	Frutinone A	65.90	0.34	*Codonopsis pilosula* (Franch.) Nannf.,<br>*Angelica sinensis* (Oliv.) Diels,<br>*Rehjnannia glutinosa* (Gaertn.) DC.
12	Taraxerol	38.40	0.77	*Codonopsis pilosula* (Franch.) Nannf.
13	Luteolin	36.16	0.25	*Codonopsis pilosula* (Franch.) Nannf.
14	Herbacetin	36.07	0.27	*Codonopsis pilosula* (Franch.) Nannf.
15	Protocatechuic acid	25.37	0.04	*Codonopsis pilosula* (Franch.) Nannf.,<br>*Angelica sinensis* (Oliv.) Diels
16	Lobetyolin	18.81	0.35	*Codonopsis pilosula* (Franch.) Nannf.
17	Rehmannioside A	25.95	0.87	*Rehjnannia glutinosa* (Gaertn.) DC.
18	Rehmaionoside C	12.89	0.34	*Rehjnannia glutinosa* (Gaertn.) DC.
19	Catalpol	5.07	0.44	*Rehjnannia glutinosa* (Gaertn.) DC.
20	Purpureaside C	3.14	0.38	*Rehjnannia glutinosa* (Gaertn.) DC.

21	Atractylenolide II	47.50	0.15	*Atractylodes macrocephala* Koidz.
22	Atractylon	41.10	0.13	*Atractylodes macrocephala* Koidz.
23	3*β*-acetoxyatractylone	40.75	0.22	*Atractylodes macrocephala* Koidz.
24	Atractylenolide I	37.37	0.15	*Atractylodes macrocephala* Koidz.
25	Atractylenolide III	31.15	0.17	*Atractylodes macrocephala* Koidz.
26	Biatractylolide	17.45	0.81	*Atractylodes macrocephala* Koidz.
27	Atractylol	-	-	*Atractylodes macrocephala* Koidz.
28	Honokiol	60.67	0.15	*Angelica sinensis* (Oliv.) Diels
29	Ferulic acid	39.56	0.06	*Angelica sinensis* (Oliv.) Diels
30	β-sitosterol	33.94	0.70	*Angelica sinensis* (Oliv.) Diels
31	Ligustilide	23.50	0.07	*Angelica sinensis* (Oliv.) Diels
32	Chlorogenic acid	11.93	0.33	*Angelica sinensis* (Oliv.) Diels

Supplementary Table 2. The related targets predicted by the major active ingredient of LBG

No.	Uniport ID	Gene names	Protein names	Degree
1	Q96RQ9	IL4I1	L-amino-acid oxidase	35
2	P54687	BCAT1	Branched-chain-amino-acid aminotransferase, cytosolic	32
3	O15382	BCAT2	Branched-chain-amino-acid aminotransferase, mitochondrial	32
4	P47712	PLA2G4A	Cytosolic phospholipase A2	32
5	Q8NCC3	LYPLA3	Group XV phospholipase A2	32
6	P15121	AKR1B1	Aldo-keto reductase family 1 member B1	31
7	P20132	SDS	L-serine dehydratase/L-threonine deaminase	27
8	P35575	G6PC	Glucose-6-phosphatase	25
9	P00439	PAH	Phenylalanine-4-hydroxylase	23
10	O60516	EIF4EBP3	Eukaryotic translation initiation factor 4E-binding protein 3	22
11	P54577	YARS	Tyrosine--tRNA ligase, cytoplasmic	21
12	P23946	CMA1	Chymase	19
13	P35557	GCK	Glucokinase	19
14	P20231	TPSB2	Tryptase beta-2	17
15	P19367	HK1	Hexokinase-1	17
16	P29218	IMPA1	Inositol monophosphatase 1	16
17	O14732	IMPA2	Inositol monophosphatase 2	16
18	P00491	NP	Purine nucleoside phosphorylase	16
19	P40939	HADHA	Trifunctional enzyme subunit alpha, mitochondrial	15
20	P30613	PKLR	Pyruvate kinase PKLR	15
21	P07195	LDHB	L-lactate dehydrogenase B chain	11
22	P09622	DLD	Dihydrolipoyl dehydrogenase, mitochondrial	6
23	P09110	ACAA1	3-ketoacyl-CoA thiolase, peroxisomal	5

24	P42765	ACAA2	3-ketoacyl-CoA thiolase, mitochondrial	5
25	Q05329	GAD2	Glutamate decarboxylase 2	4
26	P14618	PKM2	Pyruvate kinase PKM	1
27	Q9Y2Z4	YARS2	Tyrosine-tRNA ligase, mitochondrial	1
28	P32929	CTH	Cystathionine gamma-lyase	1

Supplementary Table 3. The PDB ID information of 6 targets included in BCAAs catabolism

No	Gene names	PDB ID
1	ACAA2	4C2J
2	BCAT1	2ABJ
3	BCAT2	1KT8
4	DLD	1ZMC
5	HADHA	5ZQZ
6	IL4I1	5C3L

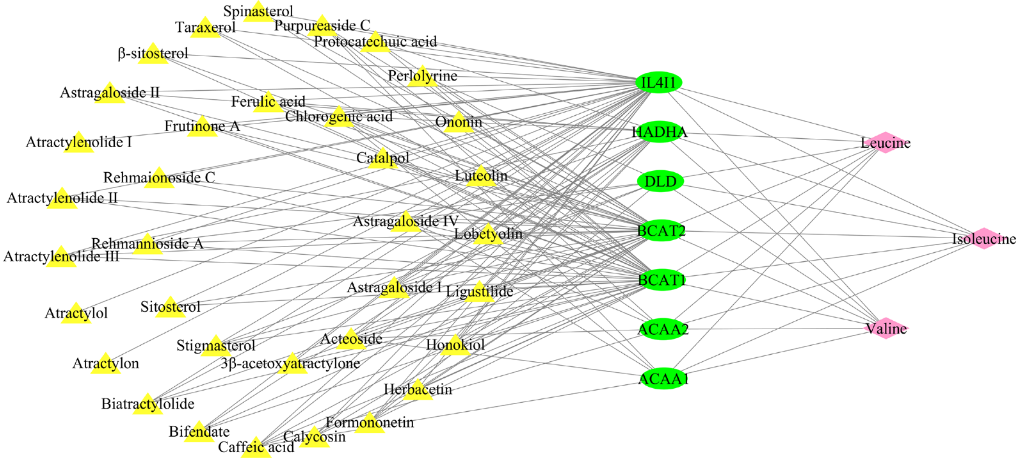

Supplementary Figure 2. "chemical components-targets-differential metabolites" regulatory network of Valine, leucine and isoleucine degradation pathway. Yellow nodes ( ) represent the chemical components, green nodes ( ) represent the targets, and pink node ( ) represent differential metabolites.

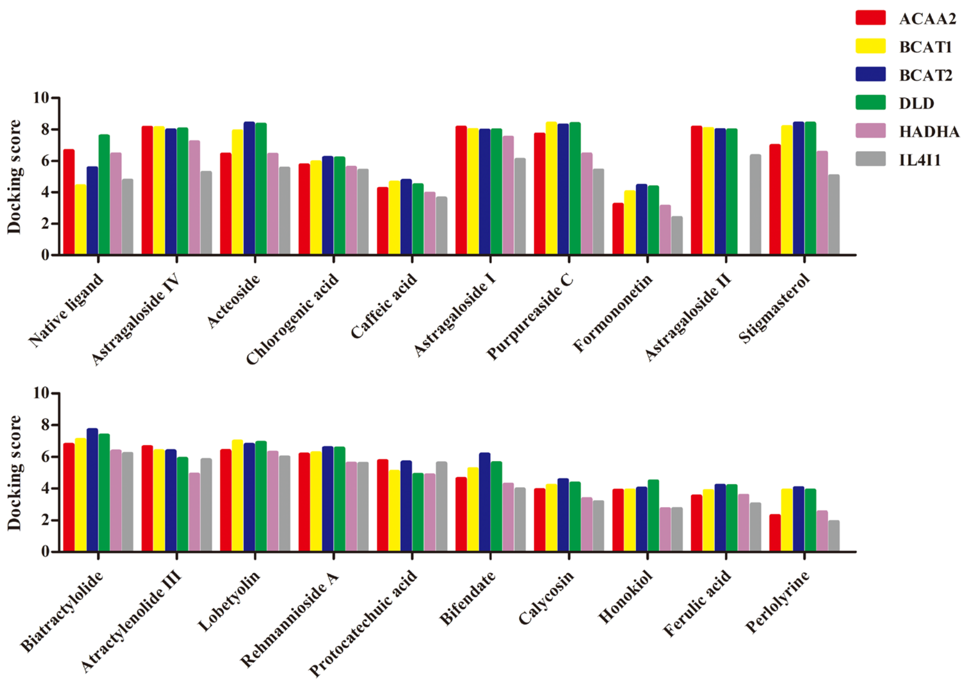

Supplementary Figure 3. 95 pairs of target-compound combinations were possessed great binding activity.
